# Enhancing Bioactivity of Hydroxyapatite Scaffolds Using Fibrous Type I Collagen

**DOI:** 10.3389/fbioe.2021.631177

**Published:** 2021-02-04

**Authors:** Paola Nitti, Sanosh Kunjalukkal Padmanabhan, Serena Cortazzi, Eleonora Stanca, Luisa Siculella, Antonio Licciulli, Christian Demitri

**Affiliations:** ^1^Biomaterials Laboratory, Department of Engineering for Innovation, University of Salento, Lecce, Italy; ^2^Laboratory of Biochemistry and Molecular Biology, Department of Biological and Environmental Sciences and Technologies, University of Salento, Lecce, Italy

**Keywords:** hydroxyapatite, magnesium, silicon, collagen, freeze - drying, bone regeneration, biodegradability

## Abstract

In the field of bone tissue regeneration, the development of osteoconductive and osteoinductive scaffolds is an open challenge. The purpose of this work was the design and characterization of composite structures made of hydroxyapatite scaffold impregnated with a collagen slurry in order to mimic the bone tissue structure. The effect of magnesium and silicon ions enhancing both mechanical and biological properties of partially substituted hydroxyapatite were evaluated and compared with that of pure hydroxyapatite. The use of an innovative freeze-drying approach was developed, in which composite scaffolds were immersed in cold water, frozen and then lyophilized, thereby creating an open-pore structure, an essential feature for tissue regeneration. The mechanical stability of bone scaffolds is very important in the first weeks of slow bone regeneration process. Therefore, the biodegradation behavior of 3D scaffolds was evaluated by incubating them for different periods of time in Tris-HCl buffer. The microstructure observation, the weight loss measurements and mechanical stability up to 28 days of incubation (particularly for HA-Mg_Coll scaffolds), revealed moderate weight loss and mechanical performances reduction due to collagen dissolution. At the same time, the presence of collagen helps to protect the ceramic structure until it degrades. These results, combined with MTT tests, confirm that HA-Mg_Coll scaffolds may be the suitable candidate for bone remodeling.

## Introduction

The use of 3-dimensional (3D) scaffolds is an approach of bone tissue engineering for the reconstruction of massive bone defects such as diseased or damaged bones (Karp et al., 2003). Scaffolds should be biocompatible and provide a temporal and spatial three-dimensional framework to form the designed tissues; also, they should act as an artificial extracellular matrix to support cell growth, proliferation and ultimately the deposition of regenerated tissue and simultaneously supply structural support to the newly formed tissue (O'Brien et al., 2007). To perform this function, scaffolds should have similar mechanical properties to those of bone repair site, biocompatibility, biodegradability, pore size between 200 and 800 μm (for bone tissue) and an open and interconnected pore structure (with a porosity >90%) (Jones, 2013). The high porosity allows supplying adequate amount of nutrients, to dispose of metabolic waste by flow transportation and to achieve tissue regeneration through tissue in-growth and then vascularization (Freed et al., 1994).

The native bone tissue consists of an organic component made mostly of collagen and an inorganic crystalline mineral component such as hydroxyapatite (HA) (Hartwig, 1990). Organic components provide flexibility, whereas inorganic components ensure strength and toughness (Wang et al., 2016). Collagen-based scaffolds are widely used because of the collagen bioactivity, which offers excellent biological performance (Salvatore et al., 2018). These scaffolds exhibited high porosity and permeability; nevertheless, they also showed poor mechanical properties and rapid enzymatic degradation, thus limiting their use when high mechanical strength is required (Akkouch et al., 2011). Therefore, due to its favorable biological properties, Collagen (Coll) could be mixed with other material such as HA to improve its mechanical properties. HA is both biocompatible and osteoconductive, although poor resorbability and brittle constructs are problems that occur when using micron-sized HA particles (Wei and Ma, 2004). One strategy to enhance resorbability is doping HA powder with biological active ions. Under *in vivo* conditions, the bone apatite has a crystalline structure allowing the substitution of constitutive bone ions (Ca^2+^, ${\text{PO}}_{4}^{3-}$, and OH^−^) with other ions present in natural bone tissue (such as Na^+^, Zn^2+^, ${\text{CO}}_{3}^{2-}$, Mg^2+^, ${\text{SiO}}_{4}^{4-}$) (Takata et al., 2005). These substitutions cause changes in crystallinity, solubility (Farzadi et al., 2014) and in bone homeostasis driving osteogenesis, angiogenesis, degradation dynamics, osteoclastogenesis, etc. (Bose et al., 2013, Hoppe et al., 2011). Starting from this evidence, more scientific works reported the fabrication of ions doped apatite (Aina et al., 2012; Padmanabhan et al., 2014; Scalera et al., 2017; Rasskazova et al., 2019; Scalera et al., 2020). Among ions found in the natural bone tissues, magnesium and silicon play an important role in the development of new bone tissue, allowing the control of bioresorption and facilitating the biomineralization and the formation of bone stock on the surface of the material (Landi et al., 2008; Munir et al., 2011).

Based on these considerations, this paper focuses on the development of novel composite scaffolds for bone regeneration using the combination of two major constituents of native bone: collagen type I and hydroxyapatite, to alleviate the problems encountered with Coll alone and HA alone (Soriente et al., 2018). Furthermore, to improve the osseointegration, bioceramic scaffolds with an open and interconnected porosity > 90% were synthesized using polymer sponge replica method (Gervaso et al., 2011), using Hydroxyapatite (HA), magnesium doped HA (HA-Mg) and silicon doped HA (HA-Si). Subsequently, to increase the bioactivity, these scaffolds were impregnated with a collagen matrix and freeze-dried, using a new approach that led to the formation of open pores on composite scaffolds surface. Therefore, the two freeze-drying approaches (new and traditional) and three composite scaffold types (HA-Coll, HA-Mg_Coll, HA-Si_Coll) were compared from a morphological and mechanical point of view. The degradation behavior of bone scaffolds is crucial for cell growth, host response and tissue regeneration (Mikos et al., 1998). Ideal scaffolds should have a degradation rate matching the regeneration rate of new bone tissue (Gervaso et al., 2016). For this reason, the scaffolds stability in aqueous buffer that simulates physiological condition was evaluated, measuring the weight loss, analyzing morphology and mechanical stability. Moreover, preliminary biological tests were assessed to demonstrate favorable cell-material interactions implying physiological responses in terms of viability and proliferation.

## Materials and Methods

### Pure and Doped Hydroxyapatite Synthesis

Pure and substituted (magnesium and silicon) HA were synthesized by aqueous precipitation reaction using Ca(NO_3_)_2_·4H_2_O, H_3_PO_4_ (85w/v), Mg(NO_3_)_2_. 6.H_2_O, Si(CH_3_CH_2_O)_4_ (TEOS) and NH_4_OH precursors. The amounts of reactants were calculated on the assumption that calcium would be substituted by magnesium and phosphorus would be substituted by silicon. Pure HA powder was synthesized by dissolving proper amounts of Ca(NO_3_)_2_·4H_2_O and H_3_PO_4_ in distilled water separately and then slowly adding Ca(NO_3_)_2_·4H_2_O solution to H_3_PO_4_ solution while stirring. In solution, Ca/P ratio fixed at 1.67. After mixing, NH_4_OH was added until pH reached a value of 10. The solution was kept on stirring for 2 h and then transferred to a borosilicate glassware and the liquid part was slowly evaporated by heating up to 400°C in a ventilated oven. After drying, the powder lumps were crushed in a ball mill and calcined to 900°C in an electric furnace to get the final powder.

For magnesium and silicon substituted HA, two compositions were prepared with nominal formula Ca_(10−*x*)_Mg_x_(PO_4_)_6_(OH)_2_, with *x* = 0.4 and Ca_10_(PO_4_)_6−*y*_(SiO_4_)_*y*_(OH)_2−*y*_, with *y* = 0.7 for nano-Mg-HA and nano-Si-HA samples, respectively. For Mg-HA synthesis, appropriate amounts of Ca(NO_3_)_2_·4H_2_O and Mg(NO_3_)_2_. 6.H_2_O were dissolved in water and added to H_3_PO_4_ solution and precipitated using NH_4_OH. For silicon substituted HA, firstly, appropriate amount of TEOS was hydrolyzed in distilled water using few drops of HCl and this silica sol was added to H_3_PO_4_ solution. Ca(NO_3_)_2_·4H_2_O solution was added to the phosphorous/ silica mixture and the pH was adjusted to 10 by adding NH_4_OH. After precipitating, pure HA synthesis procedure was carried out to obtain the final powders.

### X-ray Diffraction Analysis

The crystallinity of Hydroxyapatite calcinated powders was evaluated by X-ray diffraction (XRD). XRD analysis was performed on calcined powders using a D-Max/Ultima diffractometer (Rigaku, Tokyo, Japan). The particles crystallite size (D) was calculated from the Scherrer equation applied to the diffractogram (Sanosh et al., 2009):

(1) $$\begin{array}{l} D=\;\frac{0.89\;\lambda }{\left(\beta \cos\theta \right)} \end{array}$$

where λ is the wavelength (Cu Kα), β is the full width at the half-maximum of the HA (2 1 1) line and θ is the diffraction angle. The percentage of secondary phases in HA-Mg powder was evaluated according to the following equation (Sanosh et al., 2009):

(2) $$\begin{array}{l} \nu \text{ secondary phase }=\frac{\;\left(I_{1}+\;I_{2}\right)\;}{\left(I_{HA}+I_{1}\;+\;I_{2}\right)} \end{array}$$

were I_1_ and I_2_ represent the intensity of highest peaks present in secondary phases while I_HA_ is the intensity of the highest peak of HA.

### Scaffolds Fabrication

The Hydroxyapatite powders synthesized by precipitation method were used to obtain bioceramic porous scaffold by polyurethane sponge replica method. Three different slurries were made with the three types of apatite powders (HA, HA-Mg, HA-Si). In all slurries, the powder was added up to a final concentration of 70 wt% and polyvinyl alcohol (PVA) was used as binder at 1%wt. A poly electrolyte (Dolapix CE64, Zschimmer-Schwarz, Germany) was used as deflocculating agent and the mixture was milled in a planetary mill using zirconia balls to get a suitable ceramic suspension for infiltration. The polyurethane (PU) sponge cubes (1 cm^3^, density 30 kg/m^3^) were immersed in the slurry for impregnation, gently squeezed to remove the excess suspension and dried at 60°C overnight. The infiltrated sponges were heated to 500°C for 1 h (heating rate at 0.5°C/min) in order to burnout the polyurethane foam, following the sintering phase at 1,300°C for 3 h (heating rate at 3°C/min) and the cooling phase at room temperature (4°C/min).

A collagen slurry (0.5 % w/v) was prepared by dissolving equine tendon collagen type I (kindly provide by Typeone srl, Lecce, IT) (Raucci et al., 2019; Salvatore et al., 2020) in aqueous acetic acid solution at pH 3.8 and impregnated with hydroxyapatite scaffolds under vacuum. The impregnated scaffolds were prepared for freeze-drying (LIO-5P, Cinquepascal, Italy) using different approaches: (i) traditional method in which samples were cooled to −20°C and (ii) innovative method, in which scaffolds were placed on ice plates, immersed in cold distilled water (0°C) and cooled to −20°C.

For comparison, neat collagen scaffolds were also prepared. Briefly, the collagen suspension was mixed by a magnetic stirrer for 2 h, and then the resulting slurry was poured into an aluminum multi-well (single well diameter = 8 mm), frozen at −20°C and then lyophilized.

After lyophilizing, all scaffolds (Coll, HA_Coll, HA-Mg_Coll, HA-Si_Coll) were dehydrothermally (DHT) crosslinked (under vacuum) for 24 h at 121°C.

### Scaffold Characterization

#### Surface Morphology and Composition

The morphology of the scaffolds was studied using scanning electron microscopy (SEM) (SEM EVO^®^ 40, Carl Zeiss AG, Oberkochen, Germany) with an accelerating voltage of 20 kV. SEM micrographs were then processed and analyzed with ImageJ 1.50c. software (National Institute of Health, USA; http://rsb.info.nih.gov/ij) to determine the pore diameter by taking the average values from 20 measurements chosen randomly in the images of each sample. The diameters were reported as mean ± SEM (standard error of the mean).

Additionally, doping of Mg and Si ions in the substituted hydroxyapatite scaffolds were analyzed using Micro XRF spectrometer (Bruker M4 Tornado, Berlin, Germany).

#### Mechanical Properties

The mechanical properties of the scaffolds were evaluated by compression tests using a universal testing machine (Lloyd LR5K instrument, Fareham Hants, UK), equipped with a 1 kN load cell. The scaffolds were immersed for 2 h in PBS 1X at room temperature prior testing (T_0_). The thickness, length and width of hydrated cube specimens were measured (~1 cm^3^). The samples were placed between two parafilm layers and compressed at a crosshead speed of 0.5 mm/min. The samples (*n* = 6) from each batch of scaffolds were tested to obtain stress at failure (σ_max_), calculated as the ratio between the maximum fracture load reached and the cross-sectional area of the scaffolds. The results were expressed as mean ± SEM (standard error of the mean).

#### Stability Test

The physical integrity of composite scaffolds in simulated physiological conditions was evaluated by soaking the samples (*n* = 6) from each batch of scaffolds in 50 mL of TRIS-HCl buffer (Trizma base 0.05 M, NaCl 0.15 M, Sodium azide 0.01% w/v, pH 7.4) at 37°C (Julabo GmbH, Seelbach, Germany). The pH of the solution was maintained at 7.4 by adding 1 M HCl. At scheduled time intervals (3, 7, 14, and 28 days), the samples were recovered and gently washed with water and ethanol several times before drying. All samples were dried in oven at 60°C for 24 h and then weighed (Gervaso et al., 2016). The weight loss percentages were calculated as

(3) $$\begin{array}{l} \%\;Weight\;loss=\frac{W_{i}-W_{f}}{W_{i}} \end{array}$$

where *W*_*i*_ is the initial weight of sample and *W*_*f*_ the final weight of sample after soaking in Tris solution. The results were expressed as mean ± SEM (standard error of the mean). Moreover, post-immersion morphology of the scaffolds was qualitatively analyzed by SEM, and compression tests in wet conditions were carried out to verify possible changes in mechanical properties.

#### Cell Culture and Proliferation Assay

Human Bone Marrow-Derived Mesenchymal Stem Cells (BMSC) (ATCC-PCS-500-012, Milan, Italy) were cultured in Mesenchymal Stem Cell Basal Medium (BM) supplemented with 7% FBS, 100 IU/mL penicillin/streptomycin, 2.4 mM, 125 pg/mL FGF-b and 15 ng/mL IGF-1, at a density of 5 × 10^3^ cells/cm^2^ and incubated for 24 h at 37°C under 5% CO_2_. BMSC were used between the third and the sixth passages. Scaffolds were sterilized under UV light overnight, followed by 75% ethanol for 1 h, washed with PBS for 1 h and then incubated with culture medium overnight. Cells were seeded on the top of each scaffold at a density of 1 × 10^4^ cells per scaffold in a final volume of 50 μL. After 1.5 h, the culture medium was added to cover the scaffolds. The medium was changed every 3 days. Cell proliferation was determined using the 3-(4, 5-dimethylthiazolyl-2)-2,5-diphenyltetrazolium bromide (MTT) assay at different time points. MTT is a commonly used method to evaluate the presence of metabolically viable cells, based on the ability of viable cells to convert MTT, a soluble tetrazolium salt, into an insoluble formazan precipitate, which is quantified spectrophotometrically. Briefly, the scaffolds were first transferred into a new 24-well plate. Then 0.5 mL of culture medium containing 50 μl of MTT stock solution, 5 mg/mL of phosphate-buffered saline (PBS) solution were added to each well. After 2 h incubation, the MTT solution was removed, and 0.5 mL of 0.01 N HCl in isopropyl alcohol were added to solubilize formazan crystals. Absorbance was measured at 570 nm through a spectrophotometer.

### Statistical Analysis

For proliferation assay and compression test, values were expressed as mean ± SD for the indicated number of experiments. Differences between two groups were settled by unpaired Student's *t* test. In all comparisons, *P* < 0.05 was considered as statistically significant.

## Results and Discussion

### Evaluation of Ceramic Powders Crystallinity

**Figure 1:**
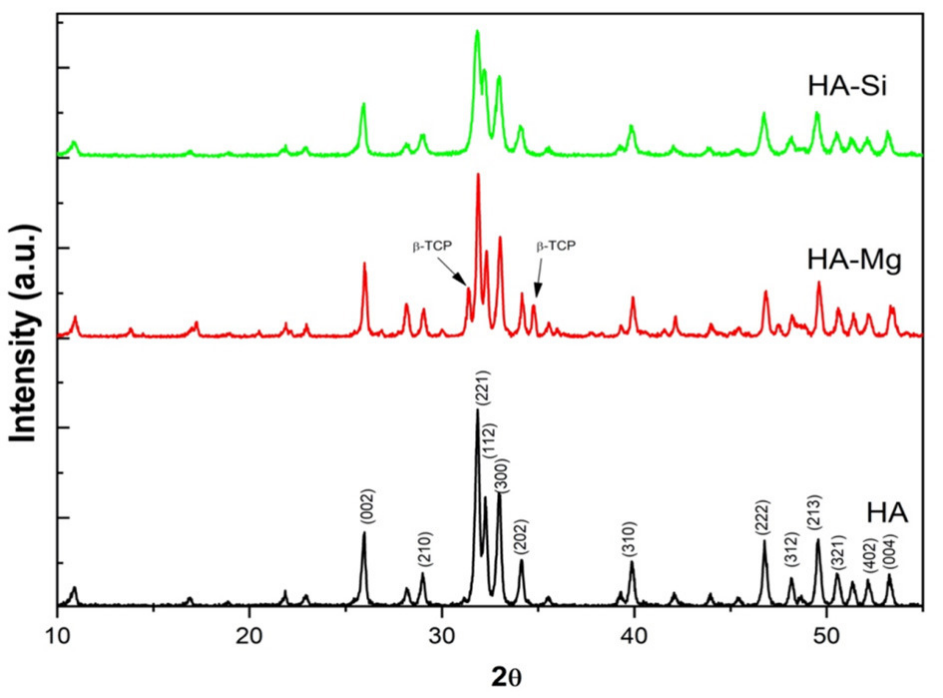
The XRD spectrum of calcined powders.

The XRD analysis (Figure 1) reveals the differences in crystallite size, crystallinity and different phases present in powders synthesized (Palazzo et al., 2011). Pure HA and HA-Mg shows a mean crystallite size of 50–60 nm, whereas HA-Si shows crystallite size of 25 nm. Pure HA shows a crystallinity around 89%. Mg doping in HA crystals resulted in an increase in crystallinity of 91% whereas Si doping decreased crystallinity to 74%. However, by examining the peaks associated with secondary phase β-tricalcium phosphate (β-TCP), it has been noticed that β-TCP was formed only in HA-Mg sample. The fraction of β-TCP calculated in HA-Mg powder is around 33%. These results indicate that Mg and Si substitution in HA crystal structure affects it crystallite size, crystallinity, and phase transformation.

### Morphological and Mechanical Analysis of Composite Scaffolds

The freeze-drying is considered as a representative technique for the fabrication of porous foam-like scaffolds. The process consists of three steps: (i) freezing of the aqueous solution, (ii) primary drying to remove ice by sublimation and (iii) secondary drying to remove unfrozen or sorbed water by desorption (Zhang, 2018). The freezing step governs several critical parameters that influence the texture of the frozen matrix and, hence, the porosity and the specific surface of the final scaffold (Abdelwahed et al., 2006). Indeed, ice is used as a template in order to produce the desired template structures. A controlled freezing process is usually required in order to control the orientation, size, and morphology of the ice crystals (Zhang, 2018) and hence the porous structure. The dimensions of ice crystals are strictly correlated with the cooling rate. At lower cooling rates, few larges ice crystals are formed compared to the higher cooling rates, where more small ice crystals are formed. The size of the ice crystals determines the size of the pores in the dried matrix, indeed scaffolds produced at high temperature results in large mean pore size (O'Brien et al., 2004; Geidobler and Winter, 2013).

**Figure 2:**
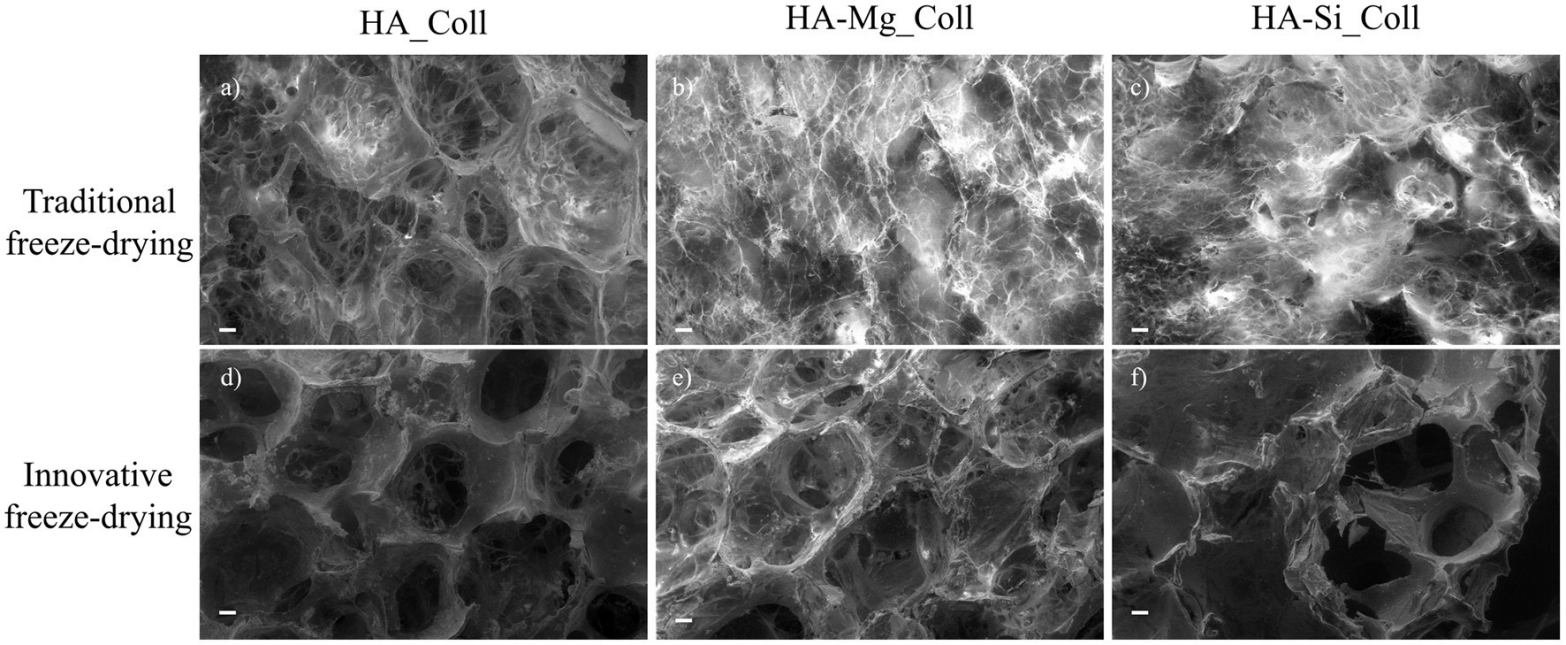
SEM images (scale bar: 200 μm) of the HA_Coll, HA-Mg_Coll, and HA-Si_Coll scaffolds freeze-dried with traditional **(a–c)** and innovative **(d–f)** approach.

**Figure 3:**
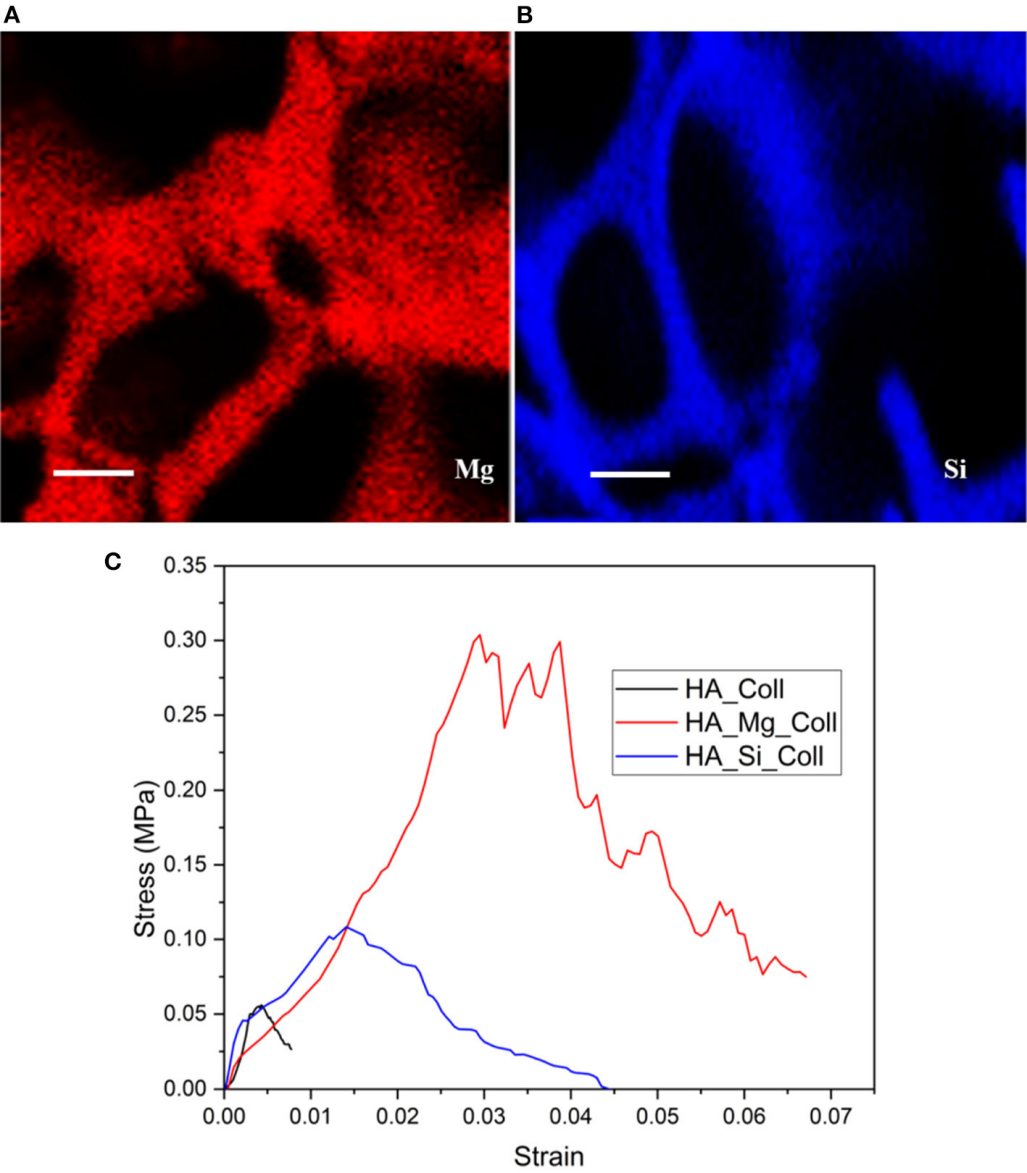
The distribution of Mg (blue signal) and Si (red signal) elements on the surface of **(A)** HA-Mg and **(B)** HA-Si ceramic scaffolds (scale bar: 100 μm) and representative stress-strain plot of composite scaffolds **(C)**.

During the crystallization of ice, a thin skin layer usually formed at the top surface of freeze-dried samples. Such skin layer may prevent the transfer of water vapor during sublimation and slow down the sublimation rate, resulting in heating the product and its fusion (Abdelwahed et al., 2006), causing the formation of closed pores at scaffolds surface. Therefore, in this work, a new approach was used. The aim of the proposed technique was to avoid the skin layer formation (which is one of the common drawbacks of this process), in order to create an open porous structure at the top surface of scaffold, thus promoting cell migration and proliferation. Using the principle in which ice is used as a template, collagen-impregnated scaffolds were first immersed in cold water and then frozen. This aims to promote the nucleation of ice crystals on the edge between the external and the internal surfaces of the samples, leading to the formation of more open and interconnected pores. Figure 2 shows the SEM images of samples prepared with traditional and innovative freeze-drying approaches. The use of this new approach seems to confirm the presence of a more open surface porosity. The formation of ice crystals on the scaffolds surface increases the presence of open pores on the outer layer of the scaffold which will come into contact with the tissues (Figures 2d–f). This represents an important advantage with respect to what is obtained with a traditional lyophilization in which collagen skin layer formed results in the absence of pores on the surface (Figures 2a–c). The open pore characteristic would promote cells migration from the outside to the inside of the scaffold and could result in an improved bone tissue growth in *in-vivo* applications. Furthermore, the pore diameters on the composite scaffolds' surface (between 200 and 600 μm) (Table 1) and average porosity around 92% of bulk ceramic scaffolds will allow cells to infiltrate, migrate and attach to the scaffold (Kramschuster and Turng, 2013). The macrostructure of bulk ceramic scaffolds exhibited open and interconnected pores and was an exact replica of PU sponge used (Queiroz et al., 2004). Elemental mapping of doped HA scaffold showed uniform distribution of Mg and Si ion in the scaffold structure (Figures 3A,B). These results proved that the doping effectively takes place during powder synthesis and remains stable during further scaffold preparation and sintering.

**Table 1:** Pore size and maxim stress at failure of collagen and composite scaffolds.

**Samples**	**Pore size (μm)**	**σ_max_ (MPa)**
Coll	110 ± 0.05	0.007 ± 0.002
HA_Coll	602 ± 0.04	0.05 ± 0.01
HA-Mg_Coll	482 ± 0.03	0.22 ± 0.07
HA-Si_Coll	524 ± 0.06	0.09 ± 0.02

The assessment of the mechanical strength of composite scaffolds was carried out through compression tests. The stress–strain compression test results are in agreement with the literature (Figure 3C) (Gervaso et al., 2011). The samples exhibit a fragile behavior in compression, typical of ceramic materials, with a slight decrease in the total stress that rapidly increases as the displacement increases. In order to evaluate the mechanical resistance of scaffolds, the maximum stress at failure (σ_max_) for all samples was evaluated. All samples showed low average stress values (Table 1), probably due to the poor compressive strength of type I collagen fibrils (0.007 ± 0.002) (Gervaso et al., 2012), because the high ratio between length and diameter makes them more unstable as the load to which they are subjected increases. However, among the three types, the HA-Mg_Coll scaffold showed the best performance, thanks to the presence of the Mg^2+^ion that enhances the sinterabilty of HA powder.

### Evaluation of Scaffolds Stability

Biodegradability is an essential feature of bioactive scaffolds, because the degradation rate must be synchronous with the bone tissue regeneration rate. Therefore, low degradation could induce an inflammatory response in the body; on the other hand, an excessively high rate will not allow the scaffold to provide a mechanical support for new tissue. To this aim, the stability of composite scaffolds in aqueous environment was evaluated and the samples were immersed in TRIS-HCl buffer (pH 7.4) at 37°C. At scheduled time intervals, i.e., after 3, 7, 14, and 28 days of soaking, stability performances of samples were checked through morphological, weight loss and mechanical evaluations (Nitti et al., 2020).

**Figure 4:**
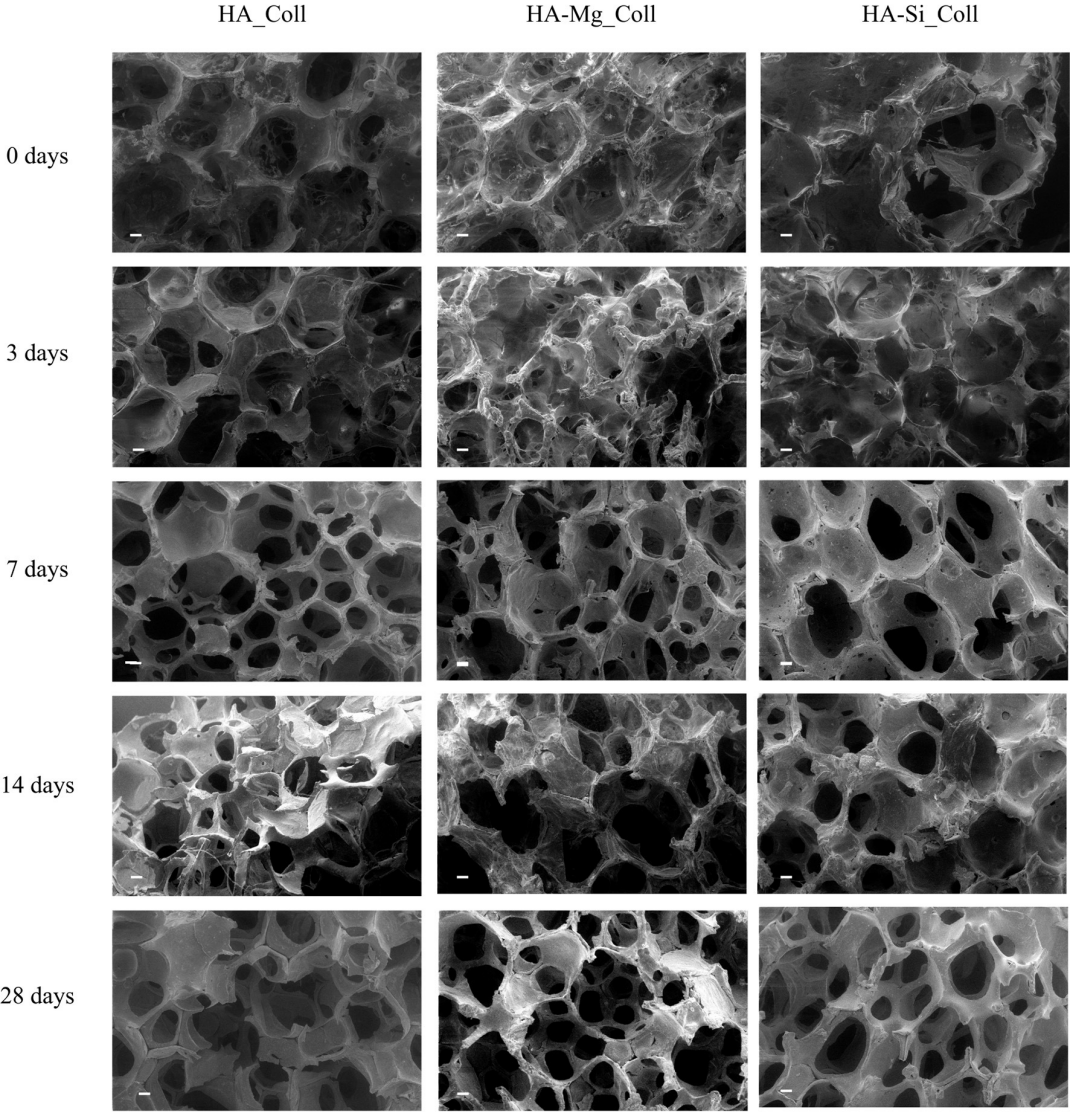
SEM micrographs (scale bar: 200 μm) of HA_Coll, HA-Mg_Coll, and HA-Si_Coll scaffolds after 0, 3, 7, 14, and 28 days in TRIS-HCl pH 7.4 at 37°C.

**Figure 5:**
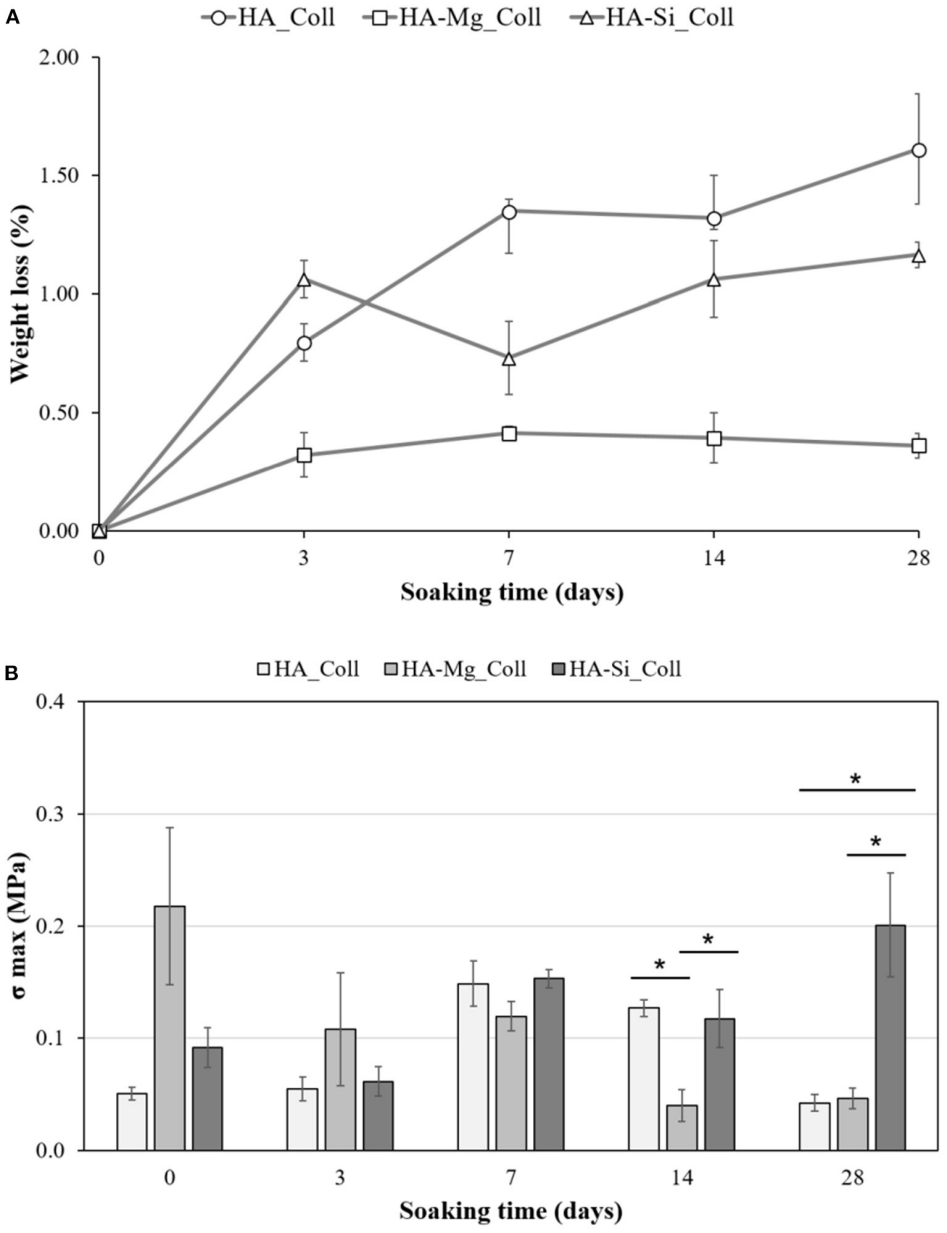
Stability test on HA_Coll, HA-Mg_Coll, and HA-Si_Coll scaffolds. Plot of weight loss % **(A)** and stress at failure (σ_max_) **(B)** after 0, 3, 7, 14, and 28 days of aging in TRIS-HCl pH 7.4 at 37°C (average values ± SEM, *n* = 6, ^*^P < 0.05).

Degradation data corresponding to neat Coll scaffolds were not reported due to the rapid rate of degradation under test conditions. The composite scaffolds after soaking, as showed in Figure 4, exhibited a gradual degradation of the collagen component. After 3 days in Tris-HCl, HA_Coll scaffolds resulted in an increased reduction of collagen at the top surface of the scaffold than the HA-Mg_Coll and HA-Si_Coll scaffolds. After a week of soaking, HA-Mg_Coll showed higher collagen dose than those in HA_Coll and in HA-Si_Coll. However, at 14 and finally at 28 days, collagen decrease continued for all scaffold types, until the collagen disappeared from the surface of the scaffold, thus showing only the ceramic component. This degradation trend was also confirmed by weight loss % measurements (Figure 5A). Samples exhibited different degradation behavior, as assessed by 28-day stability test. The HA-Mg_Coll scaffolds were almost stable in physiological conditions and revealed a significantly lower dissolution rate than HA-Coll and HA-Si_Coll scaffolds (weight loss %: 0.36 ± 0.05; 1.61 ± 0.23 and 1.17 ± 0.05 after 28 days, respectively). While for HA-Mg_Coll and HA-Si_Coll scaffolds, the percentage of weight loss remained almost the same after 3 days in solution. For the HA_Coll scaffolds, there was a first sharp increase of weight loss % after 7 days and successively after 28 days. This degradation trend of HA_Coll scaffolds was also confirmed by mechanical analysis (Figure 5B). After a week of soaking, the loss of the collagen component from the scaffolds made the ceramic component prevail, thus leading to an increase in σ_max_. After 28 days, a sharp decrease of stress at failure was present, which is similar to the observed weight loss. This is due to the initiation of degradation of the ceramic component, caused by the presence of impurities or structural defects (LeGeros, 2008). The same trend of HA_Coll scaffolds was observed in HA-Si_Coll scaffolds, although with higher σ values. During soaking, a continuous increase of stress at failure was observed due to progressive loss of collagen from the scaffold (from 0.09 ± 0.02 MPa before immersion to 0.20 ± 0.05 MPa at 28 days of soaking). However, unlike HA_Coll scaffolds at 28 day of immersion loss of ceramic component seems to be absent in Si_Coll scaffolds. Although the HA-Mg_Coll scaffolds showed better mechanical performance before immersion (0.22 ± 0.07 MPa), after immersion they presented a continuous and significant decrease of maximum stress (0.05 ± 0.01 MPa at 28 soaking days). This phenomenon could be caused by simultaneous degradation of collagen and HA-Mg due to presence of secondary phase (β-TCP) in Hydroxyapatite doped with Mg, which is more soluble and deteriorate the strength of the scaffold (Scalera et al., 2017).

### Preliminary Cell Viability Evaluation

**Figure 6:**
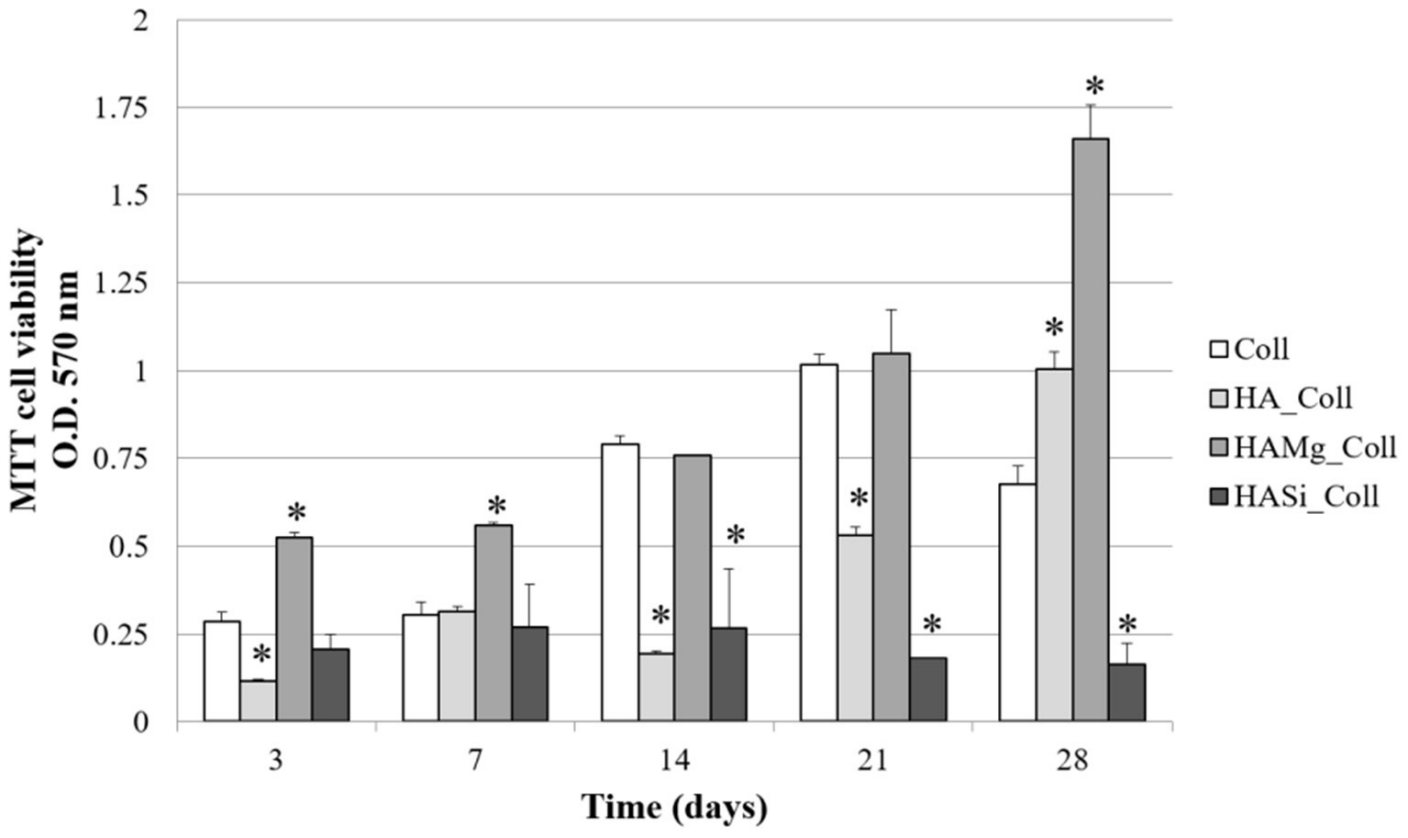
Viability of BMSC on Coll, HA_Coll, HA-Mg_Coll, and HA-Si_Coll scaffolds assessed by MTT assay after 3, 7, 14, 21, and 28 days of cell seeding. Collagen scaffolds (Coll) are used as control. Data represent the means ± SD of duplicate measurements from three independent experiments. ^*^P < 0.05.

Cell proliferation on different scaffolds was determined using MTT assay (Figure 6). Collagen scaffolds are widely used to support the growth of many cell types (Iordache et al., 2014; Salvatore et al., 2020), since they have excellent properties for tissue engineering. For this reason, collagen scaffolds were selected as positive control for BMSC proliferation (Fasolino et al., 2019). The number of viable and metabolically active BMSC increased in a time-dependent manner until 21 days in Coll scaffolds (control). HA_Coll scaffolds exhibited a gradual increase of cell proliferation throughout time but it was lower with respect to control until 21 days. After 28 days of cell seeding, BMSC viability on HA_Coll scaffolds was increased by approximately 30% with respect to control. Conversely, HA-Mg_Coll scaffolds exhibited greater cell proliferation, while HA-Si_Coll showed lower cell proliferation compared to the control. This growing increase in cell proliferation, particularly for HA-Mg_Coll scaffold could be due to the lower degradation rate, as previously reported, and also to a more stable structure during cell incubation. The superior properties of HA-Mg_Coll scaffolds are attributable to a synergic effect of collagen bioavailability at shorter time and of Mg contribution on long term incubation. This combination of factors for HA-Mg_Coll scaffolds seems to elicit a metabolic boost for cell proliferation activities compared to both the control and the other investigated samples.

## Conclusions

The development of suitable bioactive scaffolds that mimic the structure and biological characteristics of native tissue is a fundamental requirement for the treatment of injuries and diseases through a tissue engineering approach. In this study, bioactive scaffolds were fabricated successfully using a new freeze-drying approach. The proposed scaffolds presented a composite structure made with ceramic (HA, HA-Mg, and HA-Si) and collagen. The ceramic structure provides mechanical support, while collagen guarantees the biomimetic and bioactive stimuli. Porosity, degradation rate and compressive strength are fundamental aspects for the choice of a good device for bone tissue regeneration. Therefore, in this study, scaffolds with a high porosity core and open and interconnected pores on the surface were developed. When using a traditional freeze-drying approach, a collagen skin layer is present on the top surface; hence, there are no open and interconnected pores. For this reason, a new approach of lyophilisation, in which ice crystals are used as templates to create pores, was successfully developed. This resulted in open pores on top surface of the composite scaffolds, making them more suitable for the cells to colonize and migrate inside the core scaffold. Furthermore, composite scaffolds in which ceramic part was made using pure HA, HA-Mg and HA-Si were fabricated and compared, revealing best performance in terms of weight loss for HA-Mg_Coll; whereas, HA-Si_Coll scaffolds showed better mechanical resistance before and after immersion in physiological solution. However, in preliminary cell viability assays, Mg^2+^ seems to give best contribution when substituted into the structure of hydroxyapatite compared to ${\text{SiO}}_{4}^{4-}$. Mg improves the morphological, mechanical, stability properties and cell proliferation of the HA-based bioactive scaffolds, making those scaffolds suitable candidates for bone remodeling process.

## Data Availability Statement

The raw data supporting the conclusions of this article will be made available by the authors, without undue reservation.

## Author Contributions

PN: conceptualization, methodology, validation, investigation, and writing—original draft. SK: conceptualization, methodology, validation, investigation, and writing—review and editing. SC: validation and investigation. ES: methodology, validation, investigation, and writing. LS and AL: resources and supervision. CD: conceptualization, methodology, writing—review and editing, resources, and supervision. The manuscript and all the changes were written through contributions of all authors. All authors have given approval to the final version of the manuscript.

## Conflict of Interest

The authors declare that the research was conducted in the absence of any commercial or financial relationships that could be construed as a potential conflict of interest. The handling editor CG declared a past co-authorship with one of the authors, CD.
